# Landing-Takeoff Asymmetries Applied to Running Mechanics: A New Perspective for Performance

**DOI:** 10.3389/fphys.2019.00415

**Published:** 2019-04-16

**Authors:** Rodrigo Gomes da Rosa, Henrique Bianchi Oliveira, Natalia Andrea Gomeñuka, Marcos Paulo Bienert Masiero, Edson Soares da Silva, Ana Paula Janner Zanardi, Alberito Rodrigo de Carvalho, Pedro Schons, Leonardo Alexandre Peyré-Tartaruga

**Affiliations:** ^1^Laboratório de Pesquisa do Exercício, Universidade Federal do Rio Grande do Sul, Porto Alegre, Brazil; ^2^Departamento de Investigación de la Facultad de Ciencias de la Salud, Universidad Católica de las Misiones (UCAMI), Posadas, Argentina; ^3^Physical Therapy College, Universidade Estadual do Oeste do Paraná (UNIOESTE), Cascavel, Brazil

**Keywords:** kinetic, forces, spring-mass system, muscle function, biomechanics, physical endurance

## Abstract

**Background:**

Elastic bouncing is a physio-mechanical model that can elucidate running behavior in different situations, including landing and takeoff patterns and the characteristics of the muscle-tendon units during stretch and recoil in running. An increase in running speed improves the body’s elastic mechanisms. Although some measures of elastic bouncing are usually carried out, a general description of the elastic mechanism has not been explored in running performance. This study aimed to compare elastic bouncing parameters between the higher- and lower-performing athletes in a 3000 m test.

**Methods:**

Thirty-eight endurance runners (men) were divided into two groups based on 3000 m performance: the high-performance group (P_high_; *n* = 19; age: 29 ± 5 years; mass: 72.9 ± 10 kg; stature: 177 ± 8 cm; 3000_time_: 656 ± 32 s) and the low-performance group (P_low_; *n* = 19; age: 32 ± 6 years; mass: 73.9 ± 7 kg; stature: 175 ± 5 cm; 3000_time_: 751 ± 29 s). They performed three tests on different days: (i) 3000 m on a track; (ii) incremental running test; and (iii) a running biomechanical test on a treadmill at 13 different speeds from 8 to 20 km h^−1^. Performance was evaluated using the race time of the 3000 m test. The biomechanics variables included effective contact time (*t*_ce_), aerial time (*t*_ae_), positive work time (*t*_push_), negative work time (*t*_break_), step frequency (*f*_step_), and elastic system frequency (*f*_sist_), vertical displacement (*S*_v_) in *t*_ce_ and *t*_ae_ (*S*_ce_ and *S*_ae_), vertical force, and vertical stiffness were evaluated in a biomechanical submaximal test on treadmill.

**Results:**

The *t*_ae_, *f*_sist_, vertical force and stiffness were higher (*p* < 0.05) and *t*_ce_ and *f*_step_ were lower (*p* < 0.05) in P_high_, with no differences between groups in *t*_push_ and *t*_break_.

**Conclusion:**

The elastic bouncing was optimized in runners of the best performance level, demonstrating a better use of elastic components.

## Introduction

Individual differences in locomotor performance depend on specific biomechanical patterns. In bouncing terrestrial gaits, the body is idealized as a spring-mass composition thereby acting as a simple elastic system. The elastic mechanism is a well-recognized trait of human running to minimize the energy expenditure. It is known that animals, such as kangaroos (Kram and Dawson, 1998) and ostriches (Rubenson et al., 2011), make better use of the elastic mechanism and can achieve high speeds at a lower energy cost than humans. In humans, biological development and aging limit the utilization of elastic bouncing (Cavagna et al., 2008b), but this function is fully developed in adulthood, and it is related to the optimization of the use of muscle-tendon units (Legramandi et al., 2013). More than unveiling the function of single joints, the elastic model denotes whole-body aspects converging to the passive/non metabolic function of elastic energy storage and recovery (Blickhan, 1989; McMahon and Cheng, 1990). However, the role of performance level on elastic bouncing is not completely understood in distance runners.

**FIGURE 1:**
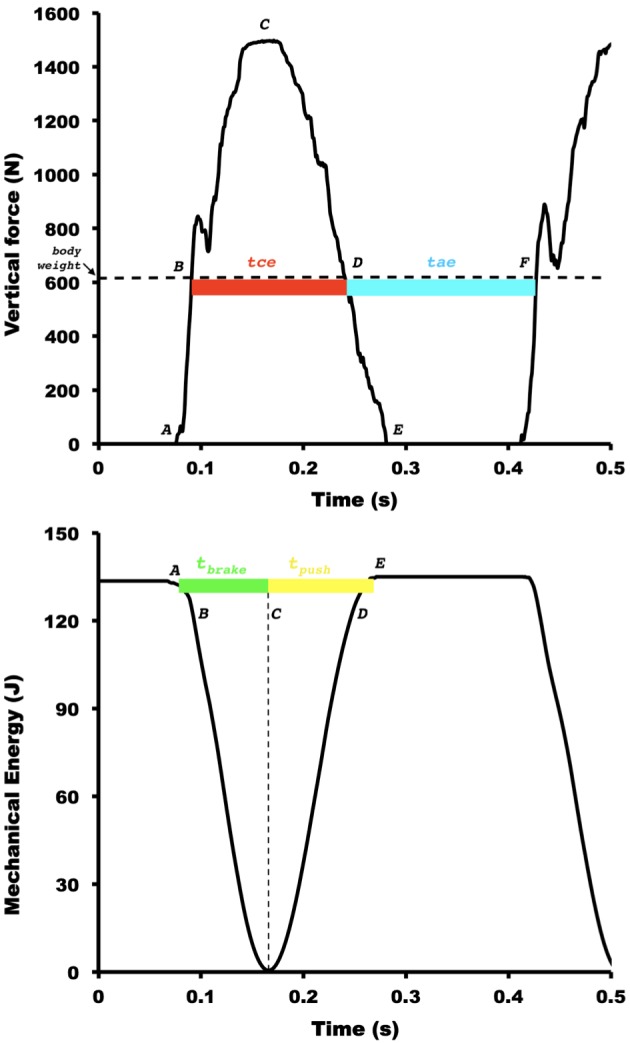
Representative figure for the vertical ground reaction force, external mechanical energy, and two main temporal asymmetries during running at 13 km h^−1^. The letters indicate the discrete points defining the main phases of spring-mass model: (*A*) landing, (*B*) downward equilibrium point (instant where body weight equals to vertical ground reaction forces (GRF) during downward trajectory of the body), (*C*) maximal vertical force and transition between negative and positive work, (*D*) upward equilibrium point (instant where body weight equals to vertical GRF during upward trajectory of the body), (*E*) takeoff, (*F*) second downward equilibrium point. The effective contact time (*t*_ce_, *B*–*D*, in red) and the effective aerial time (*t*_ae_, *D*–*F*, in blue) represent the asymmetry of rebound. The positive work time (*t*_push_, *C*–*E*, in yellow) and negative work time (*t*_brake_, *A*–*C*, in green) represent the landing-takeoff asymmetry. The horizontal dashed black line in the superior panel denotes the body weight. The vertical dashed black line in the inferior panel indicates the transition instant between negative and positive work.

The elastic function of muscle-tendon units during distance running is determined by two main asymmetries: the landing-takeoff asymmetry and the asymmetry of rebound (Cavagna, 2009). The former occurs partitioning temporally the contact time in positive and negative work of the center of body mass (*BCoM*), namely *t*_push_ (time in which mechanical energy is released at the push), and *t*_brake_ (time in which mechanical energy is absorbed at the brake), respectively (see Figure 1). The latter occurs dividing the vertical oscillation of *BCoM* during the entire step in two temporal components: the inferior vertical oscillation, called effective contact time (*t*_ce_), when the vertical force (*F_v_*) is higher than body weight is approximately equal to that of the upper part during the effective aerial time (*t*_ae_), when the vertical force on the ground is less than the body weight. Elastic bounce model is dependent on time and spatial variables. In an ideal body’s elastic bounce, the *t*_push_ equals *t*_brake_,(symmetric landing-takeoff) and the rebound is asymmetric resulting in a *t*_ae_ higher than the t_ce_ due to needed to equilibrate the vertical momentum (Cavagna, 2006). These optimized conditions take place at high speeds of human running. In a symmetric elastic system, *t*_brake_ is identical to *t*_push_. However, in slow human running, these similarities are not found because the rebound is asymmetric: specifically, at low and intermediate running speeds, the *t*_push_ is longer than *t*_brake_. Conversely, at slow speeds of human running, the *t*_push_ is longer than *t*_brake_ (asymmetric landing-takeoff), and the *t*_ce_ is identical to *t*_ae_ (symmetric rebound). In a symmetric elastic system, *t*_ce_ is identical to *t*_ae_. At high speeds, the duration of the upper part of the oscillation is higher than that of the lower part, i.e., *t*_ae_ < *t*_ce_. This phenomenon is called an asymmetric rebound (Cavagna et al., 1988). These asymmetries may be sensitive to demonstrated differences between faster and slower runners.

In addition to temporal characteristics, the asymmetry of rebound may be analyzed in spatial terms. And, again, these asymmetries are highly dependent on the horizontal speed (Blickhan, 1989). The relative amount of vertical displacement of the *BCoM* (*S*_v_) taking place at ground contact increases markedly with running speed due almost exclusively to vertical displacement during the aerial phase (Cavagna et al., 2008a; Cavagna, 2010). According to the spring-mass model, the vertical displacement of the *BCoM* is divided into *S*_ae_ and *S*_ce_, which occur during *t*_ae_ and *t*_ce_, respectively (Cavagna and Legramandi, 2015). The duration of the lower part of the oscillation represents the half-period of the bouncing system, and the *S*_v_ during this period represents the amplitude of the oscillation of the *BCoM* (Cavagna et al., 1988, 2005; Blickhan, 1989; McMahon and Cheng, 1990).

In each bounce, some of the *BCoM* mechanical energy is absorbed by muscle-tendon units during the *t*_brake_ and is successively restored through the *t*_push_. The *BCoM* vertical motion during this rebound defines the relationship between the apparent natural frequency of the bouncing system (*f*_sist_) and the step frequency (*f*_step_) at different speeds (Cavagna et al., 1988). While the contact time and step frequency are biomechanical outcomes often utilized to try explaining the running performance and economy, these variables do not reflect the integrative elastic function of muscle-tendon units (Cavagna, 2006). The *f*_sist_ and *t*_ce_, conversely, represent critical parameters of spring-mass model and, therefore, may be more closely related to running performance.

Additionally, the analysis of the ground reaction forces (GRF) improves the understanding of long-distance running performance. In elite athletes, the average peak force is correlated with performance and running economy (RE). In other research evaluating female runners, more substantial decreases in VO_2_ were associated with the highest improvements in the alignment of the resultant GRF and leg axis during propulsion. This last finding was primarily due to runners applying their resultant GRF more horizontally (Moore, 2016). On the other hand, in high-level Kenyan runners, no correlation between GRF and RE was found (Santos-Concejero et al., 2017). The sum of horizontal and vertical peak forces was found to be negatively correlated to 3000-m running performance (Støren et al., 2011). The vertical force (Fv) is a determinant of vertical stiffness (*k*_vert_), which may reflect the optimization of elastic bouncing (see section “Vertical Stiffness and System Frequency”). Therefore, trained distance runners were divided in two groups according to their performance, and analyzed for the main mechanical parameters and landing-takeoff asymmetries of spring-mass model. We hypothesized that the elastic bouncing parameters should be more optimized in the faster runners group, i.e., that faster distance runners would have a more asymmetric *t*_ae_/*t*_ce_ relation, higher *F*_v_, *k*_vert_, and *f*_sist_ than slower runners.

## Materials and Methods

### Subjects and Ethics Statement

The experiments were conducted on 38 men runners (Table 1). Inclusion criteria were minimum age of 18 years and maximum of 40 years, minimum training time of 2 years, minimum weekly training of 20 km. The exclusion criteria were: injury or illness that precludes or impairs the practice of running in the last 2 years, use of medication that affects running performance and being a smoker. The runners were divided into two groups (*n* = 19 in each group), separated by the median performance in the 3000 m test, the high-performance group (P_high_) with time 682 ± 56 s and average speed 16.5 ± 0.9 km.h^−1^ and the low- performance group (P_low_) with time 765 ± 45 s and average speed 14.9 ± 1.0 km.h^−1^. All runners were trained by professional coaches. The trained only distance running without cross-training programs (e.g., plyometrics, core, etc). The runners trained commonly on overground/outdoor environment, and, however, they were habituated to run on treadmills. The runners were classified as level 3 (trained) for P_low_ and level 4 (highly trained) for P_high_ in accordance with the guidelines proposed by De Pauw et al. (2013). Further, the organization of groups followed the aforementioned guidelines. The institutional ethics committee (No. 1.946.049 of the Universidade Federal do Rio Grande do Sul, Brazil) approved this study and the procedures conformed to the latest revision of the Declaration of Helsinki. All participants were aware of the potential risks and discomforts associated with this study before signing the informed consent form.

**Table 1:** Means and standard deviation of sample characterization for high- and low-performance groups.

Variables	P_high_ (*n* = 19)	P_low_ (*n* = 19)	Cohen’s d	*F*	*p*
Age (years)	29.0 ± 5.4	31.7 ± 6.5	−0.55	2.668	0.111
Body mass (kg)	72.9 ± 10.1	73.9 ± 7.4	−0.20	0.371	0.546
Height (cm)	177.2 ± 7.9	175.2 ± 4.8	0.17	0.274	0.604
Practice time (years)	3.7 ± 1.4	3.6 ± 1.8	0.15	0.220	0.642
Training volume/Week (km)	^∗^41.0 ± 5.2	38.1 ± 4.9	0.72	4.848	0.033
vVO_2peak_ (km⋅h^−1^)	^∗^18.9 ± 1.2	17.7 ± 0.8	1.28	13.778	0.001
VO_2peak_ (mL⋅kg^−1^⋅min^−1^)	^∗^65.5 ± 7.5	60.7 ± 6.5	0.70	4.563	0.04
3000 time (s)	^∗^656 ± 32	751 ± 29	−1.68	90.582	0.001

*The ^∗^ represents significant difference between the groups.*

### Design

All athletes performed three tests on different days with a minimum interval of 24 h between them. On the first day, an incremental maximal running test was performed. The breath-by-breath oxygen consumption (*VO*_2_) and carbon dioxide were continuously measured using a telemetric portable gas analyzer (K5, Cosmed, Rome, Italy) attached to a computer, and the heart rate was measured using a cardiac monitor (Cosmed, Rome, Italy). On the second day, the 3000 m performance test was carried out in a outdoor athletic track (SportFlex Super X, Mondo, Italy). On the third day, the athletes performed the biomechanical submaximal running on a treadmill instrumented with force sensors at different speeds. To calculate the spring-mass-model components, Cavagna’s methods were utilized (Cavagna et al., 1997, 2008b).

### Incremental Running Test

After 3 min of warm-up at 8–8.5 km.h^−1^, athletes started the protocol at 9 km.h^−1^ with a fixed treadmill grade of 1% (Jones and Doust, 1996). After each 25-s interval, the speed was increased by 0.3 km.h^−1^ until volunteers reached exhaustion. Athletes were encouraged to continue for as long as possible. After exhaustion, the athletes underwent a 5-min recovery protocol (Lourenço et al., 2011).

### 3000 m Test Performance

The 3000 m test was performed on an official athletics track. The athlete had 10 min to warm-up, including jogging and running on the track and free stretching within the given time. Two experienced researchers were at the start and the finish line of the 3000 m to record the time. The athlete was verbally encouraged to perform his best.

### Biomechanical Running Test

The athletes performed a 10-min warm-up on the treadmill at a speed of 9–10 km.h^−1^. The athletes ran at least 45 if at most 120 s according to the speed of the test (8, 9, 10, 11, 12, 13, 14, 15, 16, 17, 18, 19, and 20 km.h^−1^) and the kinetic data were recorded during the final 20 s of the each trial. The interval between each test was at least 2 min or until the athlete achieved full recovery on the Total Quality Recovery scale (Kentta and Hassmen, 1998) in order to avoid possible effects of fatigue (Fischer et al., 2015). The order of the tests was determined by simple randomization^1^.

### Data Acquisition of Ground Reaction Forces

An instrumented treadmill (super ATL model, Inbramed, Porto Alegre, Brazil) with four three dimensional load cell was used for data collection. The sensor had a low-pass and second-order filter with a cut-off frequency of 30 Hz. The data was collected with at 1000 Hz per canal with Instor software (Porto Alegre, Brazil) and a custom LabVIEW system (National Instruments, Austin, United States), and the signal was proportional to the total force collected by sensors in a vertical direction. We used the vertical component of the GRF because the spring-mass model idealized for human running (Blickhan, 1989; McMahon and Cheng, 1990) applies only the vertical GRF. Data acquisition and analysis were performed via a dedicated DAQ board and custom LabView software (National Instruments, Austin, United States). Before each acquisition, the system was calibrated to the equipment standard. There were 13 acquisitions in total, one of each speed performed by the athlete. The vertical velocities (*V*_v_) of the *BCoM* were obtained as follows.

### Landing Takeoff, Vertical Displacement, and Step Length

Ten steps of each speed were selected for analysis. The brake and push durations, respectively, *t*_brake_, and *t*_push_ (see the Figure 1) were calculated as the time intervals in which the dE_cm_ (t)/d*t* signals were below (for *t*_brake_), and above (for *t*_push_) of zero. The time interval where the d*E*_cm_ (t)/d*t* signal ≈ zero was considered the aerial time (Cavagna, 2006).

The step period and the vertical oscillation *S*_v_ of the *BCoM* were divided in two parts: a lower part, which occurred when the vertical force measured by the force platform was greater than the body weight (*t*_ce_ and *S*_ce_), and an upper part, which occurred when the vertical force was smaller than body weight (*t*_ae_ and *S*_ae_, Figure 1). The step period and the vertical displacement were also divided into the fractions taking place during the ground contact time (*t*_c_ and *S*_c_) and during the aerial time (*t*_a_ and *S*_a_). The measurement procedure and physical meaning of the *S*_v_ fractions have been described previously (Cavagna et al., 2008b; Cavagna, 2010). The step length (*L*) was calculated by multiplying the duration of the step by the average forward velocity (Cavagna et al., 1988, 2008a).

The vertical force, *F*_v_, during the stance phase is *F*_v_ = body weight + *M*_b_ *a_v_*, where *a_v_* is the vertical acceleration of the *BCoM*, i.e., the time derivative of its vertical velocity, *V*_v_. When the *V*_v_ and *E*_kv_ (0.5 *M*_b_ *V*_v_^2^) are at a maximum, the derivative is nil, *a_v_* = 0, and as a consequence *F*_v_ = body weight. The locations of the *E*_kv_ peaks attained during the step were therefore used to determine the instants where the vertical force equaled the body weight (Cavagna et al., 2008b).

### Vertical Stiffness and System Frequency

The mass-specific vertical stiffness, *k*/*M*_b_, is given by the slope of the relationship between vertical acceleration (*a_v_*) and *S*_v_ in the range corresponding to the amplitude of the oscillation of the spring-mass system, i.e., from its equilibrium position (*a_v_* = 0) to its maximal deformation *a_v_*_,mx_; (Cavagna et al., 1988). The mass-specific vertical stiffness was therefore measured as *k*/*M*_b_ = *a_v_*_,mx_/*S*_ce_, where *S*_ce_ is the amplitude of the oscillation, i.e., the vertical displacement of the *BCoM* from *a_v_* = 0 to *a_v_*_,mx_. Correspondingly, the natural frequency of the spring–mass system was calculated as *f*_sist_ = 1/(2*t*_ce_) = (*k*/Mb)0.5/(2π).

### Statistical Analysis

The statistical tests were performed using the SPSS 25 package (IBM Corporation, Inc., New York, United States). All descriptive statistics presented in the text, tables and figures are mean values ± SD. The level of significance was α = 0.05. Generalized linear analysis models (GLMM) were used and Bonferroni *post hoc* tests were used to find statistical differences. Initially, intra-subject variability was tested to determine candidate random variables due to the hierarchical nature in GLMM (Nakagawa and Schielzeth, 2013). The condition was not found to be a variable according to the test of compliance with the intraclass correlation coefficient (ICC-pre) of the analysis of variance components by the maximum restricted likelihood method. The pre-ICC was not higher than 5%, and none had a random effect. The Cohen’s coefficient (d) was calculated to determining the effect size between 0.2 to 0.5 to small, 0.5 to 0.8 medium and higher than 0.8 to large effect (Cohen, 2013). All individual results are shown in the Supplementary Material.

## Results

Table 1 shows the characterization of subjects. Age, body mass, height, practice time were similar between groups. The 3000 m running performance, the vVO_2peak_, and VO_2peak_ were higher in the P_high_.

**FIGURE 2:**
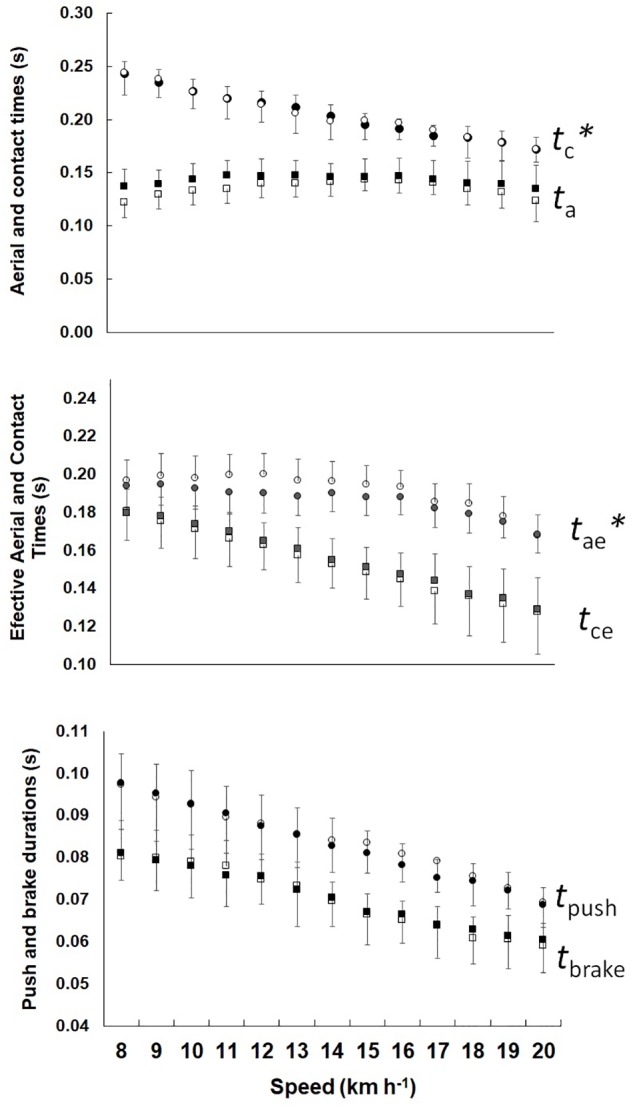
Mean and standard deviation in aerial and contact times, effective aerial, and contact times, *t*_push_ and *t*_brake_ durations plotted as a function of the speed. Left show P_high_ and right P_low_. The ^∗^ represents significant difference between the groups.

Figure 2 shows the step phase, the phases of the bouncing system and the *t*_push_, *t*_brake_ durations for P_high_ and P_low_. It was observed that *t*_a_ was higher for P_high_ (*F* = 13.987, *p* < 0.01); however, in *t*_c_ there was no difference between groups (*F* = 0.001, *p* = 0.971), while in *t*_ce_ was lower (*F* = 6.328, *p* < 0.01) and *t*_ae_ was grater (*F* = 13,987, *p* < 0.01) in P_high_. The *t*_push_ and *t*_brake_ showed no differences between groups (*F* = 2.159, *p* = 0.142 and *F* = 0.108, *p* = 0.742, respectively).

**FIGURE 3:**
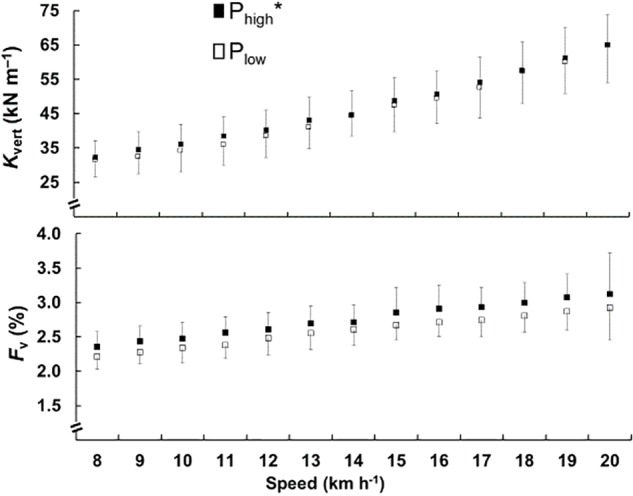
Mean and standard deviation of the vertical stiffness (*k*_vert_) in the top chart and relative vertical force (*F*_v_) in the lower chart, for vertical distance traveled, is plotted as a function of the speed. The black square represents the P_high_ and the open square represents the P_low_. The ^∗^ represents significant difference between the groups.

The *k*_vert_ and the *F*_v_ are shown in the Figure 3. The two charts are presented together because the stiffness is the ratio between *F*_v_ and *S*_ce_, and it is possible to observe their relationship. The results show that *k*_vert_ (*F* = 4.460, *p* < 0.05) and *F*_v_ (*F* = 30.824, *p* < 0.01) were greater in P_high_.

**FIGURE 4:**
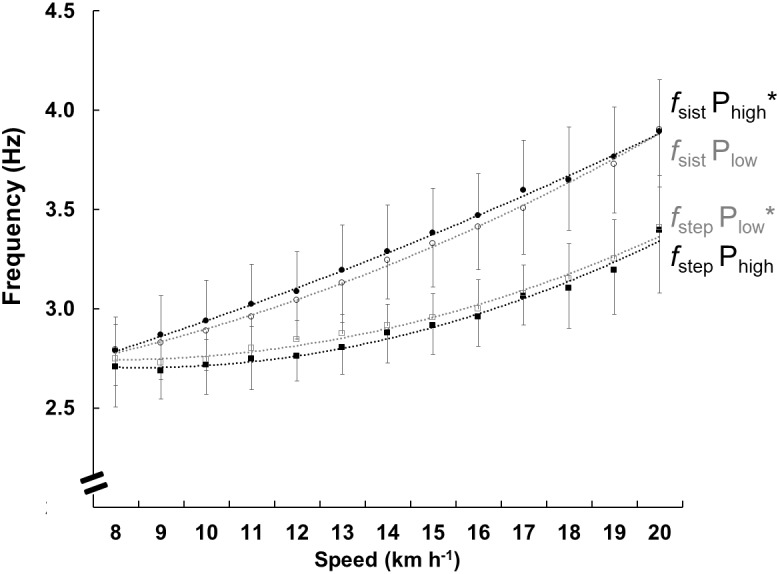
Mean and standard deviation of the frequency parameters at each speed for both groups. The black circles show the natural frequency of the system (*f*_sist_) in P_high_ and the open circles show P_low_. The black squares show the step frequency (*f*_step_) in P_high_ and the open squares show P_low_. The lines represent the polynomials of the second order function, the black color represents P_high,_ and the gray represents the P_low_. Its only purpose is to facilitate the viewing of results. The ^∗^ represents differences between the groups.

The *f*_sist_ and *f*_step_ are shown in Figure 4. The *f*_sist_ was higher in P_high,_ (*F* = 4.199, *p* < 0.05) and *f*_step_ was higher in P_low_ (*F* = 4.173, *p* < 0.01). With increasing speed, the *f*_sist_ (*F* = 96.416, *p* < 0.001) and *f*_step_ (*F* = 46.664, *p* < 0.001) increased in both groups, the difference between the two frequencies *f*_sist_ and *f*_step_ was lower in the P_high_ group.

**FIGURE 5:**
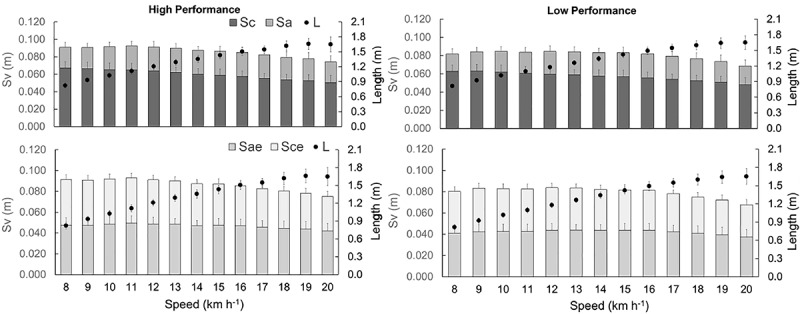
Vertical displacements of *BCoM* (*S*_v_) during contact time (*S*_c_), aerial time (*S*_a_), effective aerial time (*S*_ae_), effective contact time (*S*_ae_), and step length (*L*). The gray bars are related to the left axis and the black circles to the right axis.

Figure 5 shows the *S*_v_ components divided into *S*_a_, *S*_c_, *S*_ae_, and *S*_ce_. The *S*_a_ (*F* = 29.475, *p* < 0.01), *S*_ae_ (*F* = 83.044, *p* < 0.001), *S*_c_ (*F* = 25.835, *p* < 0.01), *S*_ce_ (*F* = 52.494, *p* < 0.001), *S*_v_ (*F* = 25.835, *p* < 0.001) and *L* (*F* = 4.548, *p* = 0.034) were higher in P_high_ than P_low_. With increasing speed, *S*_v_ was reduced (*F* = 23.268, *p* < 0.001) and *L* (*F* = 667.259, *p* > 0.001) progressively increased in both groups.

## Discussion

We compared the elastic bouncing parameters in athletes with different running performances and investigated the possible mechanical adaptations for better runners. Our hypothesis was confirmed since crucial variables of the spring-mass model were significantly better for the P_high_ group, demonstrating that the *t*_ce_/*t*_ae_ parameters are sensitive to detecting differences in the performance of athletes.

The results showed that asymmetry *t*_ce_/*t*_ae_ is more sensitive to represent the elastic mechanism than the traditional *t*_c_/*t*_a_ asymmetry, and allows one more application of the parameters of the mass-spring system in performance athletes. When analyzing only the *t*_c_, there was no difference between groups, but when analyzing *t*_ce_, it was lowest for P_high_, while *t*_ae_ was highest, showing a more effective force application in the ground with a greater *L* (see Figure 4). Future studies should consider the use of *t*_ce_ and *t*_ae_, which are more representative of the elastic system in running than values usually used in the literature (Morin et al., 2007; Pantoja et al., 2016). Furthermore, no relationship between contact time and RE were found (Santos-Concejero et al., 2017). The use of more specific parameters of the spring-mass model has an additional potential to understand the contribution and role of biomechanics in RE and performance.

Differently, *t*_push_ and *t*_brake_ did not present differences between groups, showing that the general mechanical differences are not readily enough to demonstrate integrative changes on positive and negative mechanical work duration. In Cavagna’s seminal work on landing-takeoff asymmetries, it has been shown that with increasing speed, the work contribution by the contractile machinery is gradually replaced by elastic storage and release by tendons (Cavagna, 2006). These asymmetries increase with aging (Cavagna et al., 2008b). The muscular force seems to determine the impaired elastic function in the elderly. In our study, the muscular force capabilities probably are not drastically different between the analyzed groups. On the other hand, our findings on the rebound more asymmetric (*t*_ce_ and *t*_ae_ different) in P_high_ than P_low_ are consistent with the stiffer tendon structures in the knee extensors and more compliant ones in the plantar flexors in better long distance runners (Kubo et al., 2000).

It is possible to observe that the *S*_v_ is higher in the P_high_, this behavior is a response of greater optimized elastic bouncing because of the larger *L*. The P_low_ obtained a smaller amplitude of the vertical oscillation of the *BCoM*, with a lower *F*_v_ and a reduced duration of the aerial phase, implying less elastic energy stored and a higher step frequency. Similar outcomes were observed when testing effects of step frequency (Morin et al., 2007) and comparing young and old people (Cavagna et al., 2008b).

The stiffness is the ratio of *F*_v_ to *S*_ce_. The *k*_vert_ is higher in the P_high_ due to proportionally higher *F*_v_ than *S*_ce_ in comparison to the P_low_ group. Considering that stiffness is a function of *f*_sist_, the increase in the *k*_vert_ is related to *t*_ce_ lower in the P_high_ (Cavagna et al., 2008a), corroborating the modelistic approach of elastic bouncing proposed by Blickhan (1989). The *F*_v_ is crucial for spring-mass parameter calculations and depends on the running technique, principally the *L* and *f*_step_ adapted from a more efficient speed, to select frequencies for lower oxygen consumption (Cavanagh and Williams, 1982). Interestingly, the alleged high *k*_vert_ and small tc of human running have played a critical role in the understanding of the mechanical determinants of the distance running performance (Arampatzis et al., 1999; Morin et al., 2007; Santos-Concejero et al., 2017). Nevertheless, the mechanical concept of stiffness is not equal to elasticity and, therefore, these concepts should not used equivalently. In fact, it has been shown that the elastic mechanism is impaired in old men, and the *k*_vert_ was remained unchanged (Cavagna et al., 2008a). Given the contradictory previous findings, we claim that the role of the elastic function on running performance be investigated not only by applying punctual and indirect aspects of spring-mass model (as *k*_vert_ and *t*_c_, respectively) but also including key asymmetries of spring-mass model related to the asymmetry of rebound (*t*_ce_/*t*_ae_) and landing-takeoff asymmetry (*t*_push_/*t*_brake_).

The higher running speeds contribute to increased *k*_vert_ and diminished leg stiffness (Blickhan, 1989; Arampatzis et al., 1999). During a 400-m run, *k*_vert_ starts higher and decreases throughout the race, and is also related to speed decreased (Hobara et al., 2010). It is possible to observe that in addition to speed, in P_high_ also has greater *k*_vert_, and besides the speed, the athletic level is a marker that alters the elastic bouncing. The greatest differences were found at speeds close to those that athletes ran in their 3000 m test, and at speeds from 18 km.h^−1^, *k*_vert_ values were very close. We hypothesized that athletes exhibit optimized elastic bouncing at speeds for training and competition.

At P_low_, the higher *f*_step_ than at P_high_ is due to a lower *t*_a_, and not to a higher *f*_sist_ (larger in P_high_) which means that the system was in greater vertical oscillation and greater *L*. The amplitude of the vertical oscillation is indeed reduced in old subjects, resulting in an approximately 20% smaller elastic recovery and a greater *f*_step_ (Cavagna et al., 2008a). It is worth considering that we also found dissimilarities between the groups, nevertheless, at a lower magnitude, which is an indicator that the effects of performance are less responsible than aging for these mechanical alterations on running. In other study, subjects consumed less energy when they could maintain stiffness, so that the *f*_sist_ of the model was close to the real *f*_step_ (Dalleau et al., 1998). This finding coincides with the view that P_high_ has been more elastic since *f*_sist_ = 1/2*t*_ce_. Integratively, these findings suggest that the more “elastic” may induce to a higher mechanical efficiency and economy (Peyré-Tartaruga and Coertjens, 2018). Nevertheless, that assumption remains to be tested via controlled experiment.

In conclusion, elastic bouncing is dependent on the level of performance. More trained runners presented a spring-mass system oscillating at a higher frequency and larger vertical amplitude. These responses, in turn, result in a greater stride length.

### Limitations

The main limitations of the study are related to biomechanical differences between run on treadmill versus overground, limb dominancy symmetry and size effects. There are systematic differences found in studies using force platforms on the ground versus force sensors instrumented in treadmills. For example, the tae is consistently lower in treadmill (170 ms at 20 km h^−1^) than in overground (approximately 210 ms at 20 km h^−1^, Cavagna, 2006). Possibly, a higher complacence in the treadmill should be affecting these results, particularly at high horizontal speeds where the heart rate and perceptual measures seem to be most affected (Miller et al., 2019). Thus, even not invalidating the main messages, our absolute values are restricted to treadmill running. The dominancy limb symmetry was not evaluated here, and future studies may examining whether the lateral asymmetry (dominant vs. non-dominant) of elastic mechanism influences the performance level, therefore, extending previous findings (Carpes et al., 2010; Pappas et al., 2015). And, further analysis should be performed analyzing the size effects on the elastic mechanism due to inherent role of body dimension on running mechanics and energetics, even including the allometric approach (Tartaruga et al., 2009, 2010).

## Ethics Statement

This study was carried out in accordance with the recommendations of UFRGS Committee with written informed consent from all subjects. All subjects gave written informed consent in accordance with the Declaration of Helsinki. The protocol was approved by the UFRGS Committee.

## Author Contributions

LP-T and RdR conceived of the study and designed the experiments. LP-T obtained the funding. RdR, HO, NG, MM, EdS, AZ, AdC, PS, and LP-T carried out the analysis, interpreted the statistical results, and drafted the manuscript. RdR, HO, NG, MM, EdS, AZ, AdC, and PS collected the data. All authors contributed to the manuscript writing, read, and approved the final manuscript.

## Conflict of Interest Statement

The authors declare that the research was conducted in the absence of any commercial or financial relationships that could be construed as a potential conflict of interest.

## Footnotes

1 www.randomizer.org

## Supplementary Material

TABLE S1 The table contains sheets including individual data for all variables.

## Abbreviations

*a*_v_: Vertical acceleration
*BCoM*: Center of Mass
*E*_cm_: Mechanical energy of the center of mass
*f*_sist_: Elastic system frequency
f_step_: Step frequency
*F*_v_: Vertical force
*k*_vert_: Vertical stiffness
*L*: Step length
*M*_b_: Body mass
*S*_ae_: Vertical displacement in aerial time
*S*_ce_: Vertical displacement in contact time
*S*_v_: Vertical displacement
*t*_a_: Aerial time
*t*_ae_: Effective aerial time
*t*_break_: Negative work time
*t*_c_: Contact time
*t*_ce_: Effective contact time
*t*_push_: Positive work time
*VO*_2_: Oxygen consumption
*V*_v_: Vertical velocity.
